# Low infiltration of tumor-associated macrophages in high c-Myb-expressing breast tumors

**DOI:** 10.1038/s41598-019-48051-1

**Published:** 2019-08-12

**Authors:** Nataliya Volodko, Taras Gutor, Orest Petronchak, Roman Huley, Monika Dúcka, Jan Šmarda, Lubor Borsig, Petr Beneš, Lucia Knopfová

**Affiliations:** 1 Danylo Galytsky Lviv National Medical University, Department of Oncology and Medical Radiology, Lviv, Ukraine; 2 Lviv Regional Oncological Center, Lviv, Ukraine; 3 Department of Experimental Biology, Faculty of Science, Masaryk University, Brno, Czech Republic; 4 International Clinical Research Center, Center for Biological and Cellular Engineering, St. Anne’s University Hospital, Brno, Czech Republic; 5 Institute of Physiology, University of Zurich and Zurich Center for Integrative Human Physiology, Zurich, Switzerland

**Keywords:** Breast cancer, Cancer microenvironment, Tumour biomarkers

## Abstract

Tumor-associated macrophages (TAMs) are prominent components of tumor stroma that promotes tumorigenesis. Many soluble factors participate in the deleterious cross-talk between TAMs and transformed cells; however mechanisms how tumors orchestrate their production remain relatively unexplored. c-Myb is a transcription factor recently described as a negative regulator of a specific immune signature involved in breast cancer (BC) metastasis. Here we studied whether c-Myb expression is associated with an increased presence of TAMs in human breast tumors. Tumors with high frequency of c-Myb-positive cells have lower density of CD68-positive macrophages. The negative association is reflected by inverse correlation between *MYB* and *CD68/CD163* markers at the mRNA levels in evaluated cohorts of BC patients from public databases, which was found also within the molecular subtypes. In addition, we identified potential *MYB*-regulated TAMs recruiting factors that in combination with *MYB* and *CD163* provided a valuable clinical multigene predictor for BC relapse. We propose that identified transcription program running in tumor cells with high *MYB* expression and preventing macrophage accumulation may open new venues towards TAMs targeting and BC therapy.

## Introduction

Breast cancer (BC) is the most common malignant disease in women, with one million new cases diagnosed worldwide per year. Tumors engage various components of immune system throughout their evolution. Among these components, tumor-associated macrophages (TAMs) represent a major cell population constituting up to 50% of tumor mass^1,2^. TAMs, a macrophage population recruited and educated by tumor cells, resemble M2-like macrophages^3^. Unlike M1-like macrophages exhibiting pro-inflammatory and anti-cancer functions, the M2-like macrophages are immunosuppressive cells contributing to the matrix-remodeling, angiogenesis, chemoresistance and metastasis and hence favor tumor growth and dissemination^4–9^. The direct correlation between high amount of TAMs and worse prognosis/low survival rate of breast cancer patients was demonstrated and TAMs depletion was suggested as therapeutic strategy in breast cancer^10–14^. Less known are mechanisms of attraction and polarization of TAMs inside the malignant tissue. Several soluble factors of tumor microenvironment, secreted by tumor and stromal cells, such as CCL2 (MCP1, monocyte chemoattractant protein-1; C-C motif chemokine ligand 2), CSF1 (colony-stimulating factor 1), CSF2 (colony-stimulating factor 2), VEGFA (vascular endothelial growth factor A), CCL18 (C-C motif chemokine ligand 18), CCL20 (C-C motif chemokine ligand 20), and CXCL12 (C-X-C motif chemokine ligand 12) are doubtless involved in the processes of monocytes recruitment and their polarization at the tumor sites^1,15^. However, how the cytokine production by tumor cells orchestrates the tumor microenvironment, including TAMs remains rather unclear.

The c-Myb protein encoded by *MYB* gene, is transcription regulator required for the maintenance of stem cells in bone marrow, colon epithelia, and neurogenic niches in adult brain^16^. Its expression has been linked with leukemias and epithelial cancer, most notably colon and breast cancers. c-Myb was described to have oncogenic and tumor suppressor activities in BCs^17–21^. However, clinical data have unanimously associated *MYB* overexpression with a good prognosis for BC patients^19,20,22^. Better survival rate has been linked with a lower risk of lung metastasis in patients with high *MYB* expression^23^.

Recently, we have described the functional association between Ccl2 and c-Myb in regulation of the monocyte-assisted extravasation capacity of breast tumor cells. c-Myb efficiently suppressed the inflammatory circuit including the Ccl2 chemokine in mouse models of BC and attenuated tumor dissemination suggesting that c-Myb-regulated transcriptional program may affect recruitment and/or activity of immune cells^23^. Because Ccl2 is a well-known monocyte/macrophage recruiting factor in BC^24–28^, we explored an association between c-Myb immunostaining in tumor cells and TAMs infiltration in clinical specimens of breast carcinomas. While causal interdependence between TAMs and cancer progression has been established, the molecular mechanisms linking oncogenic mutations in the tumor cells to the modulation of the microenvironment remain to be elaborated. The identification of a tumor-expressed transcription factor program in association with TAMs abundance may help to find a valuable multigene biomarker for the assessment of a clinical outcome that is superior to TAMs enumeration *per se*.

## Results

### High *MYB* levels mark tumors with low infiltration by CD68+ macrophages

**Figure 1:**
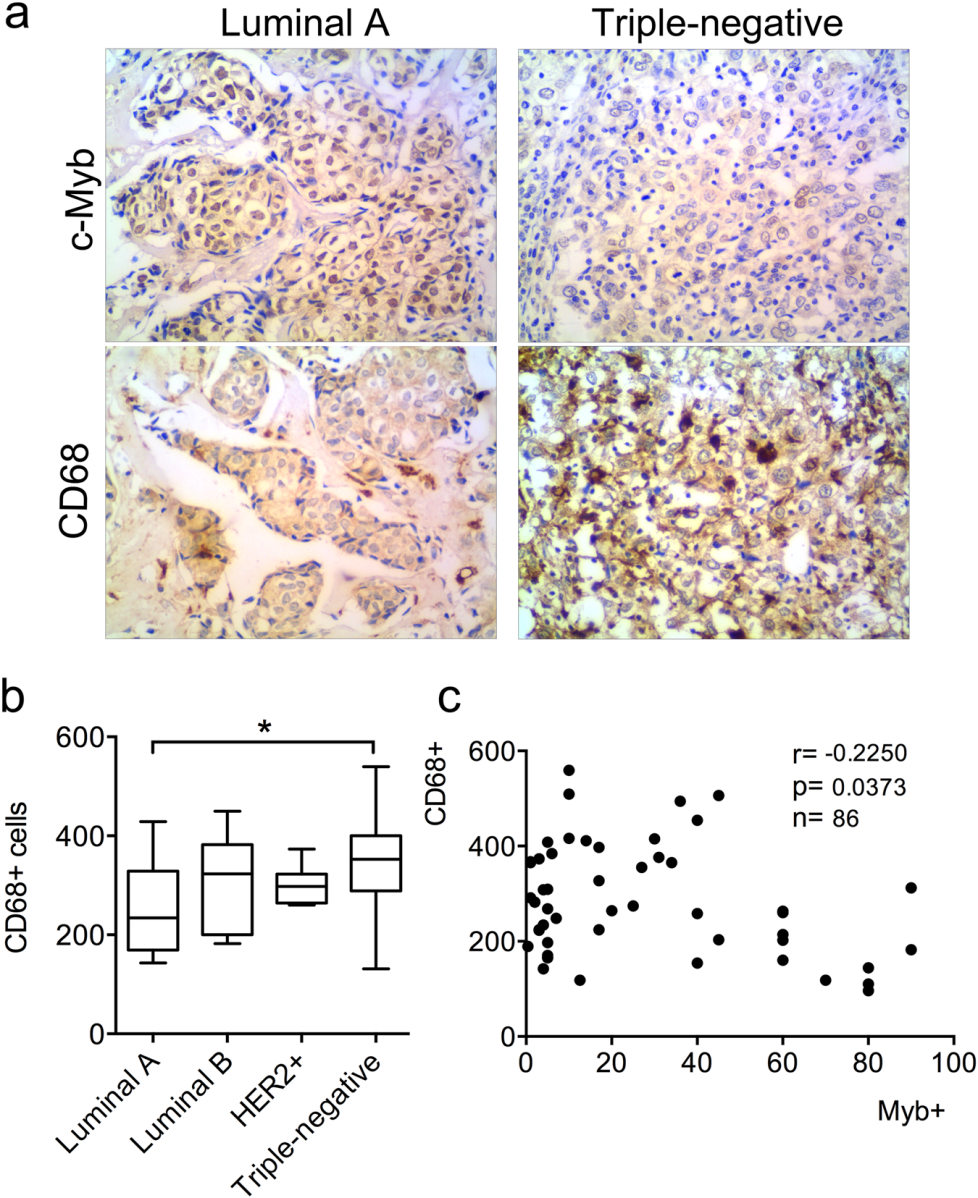
Detection of CD68+ TAMs, and c-Myb in BC patient samples according to the ER/PgR/HER2 status. (**a**) c-Myb and CD68 proteins in BC tissues were detected by IHC. Invasive ductal BCs, *left* - luminal A subtype (>50% of c-Myb+ tumor cells, low number of CD68+ cells), *right* - triple negative case (<5% of c-Myb+ tumor cells, high number of CD68+ cells). (**b**) Absolute amount of CD68+ cells in 40 high power fields X1350 in different subtypes. (**c**) Correlation between percentage of c-Myb+ tumor cells and number of CD68+ cells as determined by IHC in a cohort 86 BC patients. Pearson correlation coefficient (r), logrank p value (p) and number of patients (n) are indicated.

First, we explored the number of CD68+ cells within subgroups with different immunohistochemical (IHC) status of estrogen receptor (ER), progesteron receptor (PgR) and human epidermal growth factor receptor 2 (HER2), i.e. luminal A (ER+PgR+HER2−), luminal B (ER+PgR+HER2+), HER2+ (ER−PgR−HER2+) and triple negative (ER−PgR−HER2−). In line with published data we observed a higher number of CD68+ macrophages in triple negative subgroup (Fig. 1a,b). On the contrary, the stronger intensity of *MYB* expression and higher frequency of c-Myb-positive cells were found in ER-positive tumor samples. When patients of all subtypes (n = 86) were combined, the statistically significant inverse correlation (r = −0.23, p = 0.0373) between the amount of CD68+ cells and frequency of c-Myb+ tumor cells was revealed (Fig. 1c). The significant inverse correlation (r = −0.27, p = 0.0258) was maintained in ER-positive patients, that constitute majority (n = 66) of patients in study group, while within 20 patients of ER-negative subgroup there was no significant correlation (r = 0.199, p = 0.401) (Supplementary Fig. S1).

### *MYB* is inversely correlated with *CD163*/*CD68* mRNA in BC molecular subtypes

**Figure 2:**
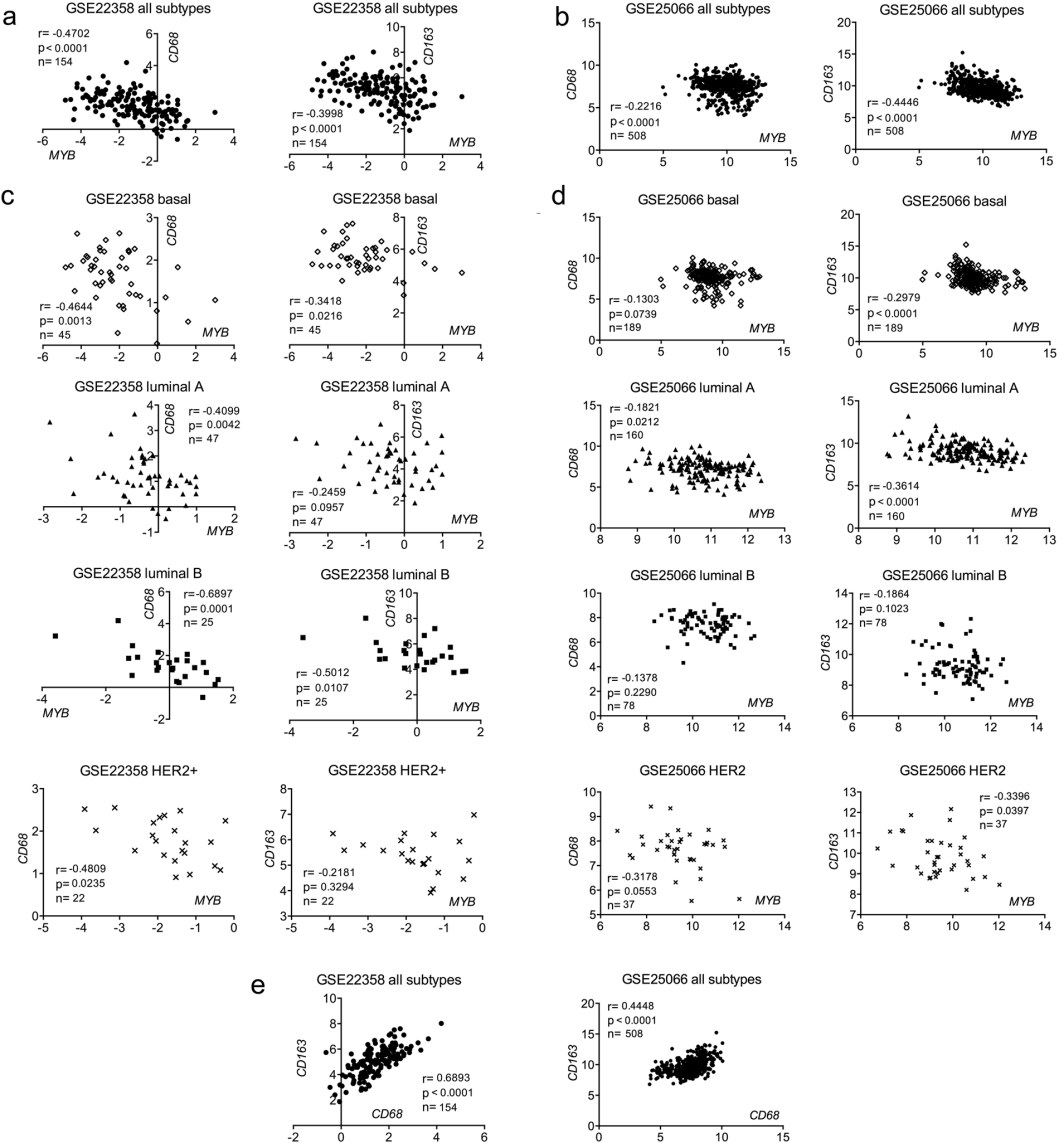
Expression of *MYB* inversely correlates with *CD68* and *CD163* mRNAs in human BCs. Two datasets were used for correlation analysis: GSE22358 (left; **a**,**c**,**e**) and GSE25066 (right; **b**,**d**,**e**). Pearson correlation coefficients (r) between mRNA expression of indicated genes (*MYB* vs *CD68*, *MYB* vs *CD163*, and *CD68* vs *CD163*), logrank p value and number of patients (n) are indicated in the graphs. Patients of all subtypes were included in **a**,**b**,**e**; and stratified according to the molecular subtypes (by the PAM50 classifier) in **c**,**d**.

We used publically available databases to investigate the transcript levels of *MYB* and two monocyte/macrophage markers *CD68* and *CD163*^29^. Medisapiens database (medisapiens.org) showed an inverse correlation between *MYB* and *CD163* mRNAs in BC patients (r = −0.192, p < 0.001, n = 1830) (Table S1). *CD68* was inversely correlated with *MYB* in breast lobular carcinomas (r = −0.282, p < 0.001, n = 83), only marginal associations were found in ductal and other carcinomas (Table S1). Then, we calculated correlations between *CD68*/*CD163* and *MYB* in a cohort of 154 BC patients (GSE22358) across and within molecular subtypes defined using the PAM50 classifier in the original study^30^. As expected, *CD68* and *CD163* mRNAs were in positive correlation (r = 0.69, p < 0.0001). *MYB* expression negatively correlated with both *CD68* (r = −0.47, p < 0.0001) and *CD163* (r = −0.4, p < 0.0001) across BC subtypes (Fig. 2a). Importantly, the inverse correlations were found also within basal, luminal A, luminal B and HER2+ subtypes, though in luminal A and HER2+ groups correlations with *CD68*, not *CD163*, were significant (Fig. 2c, Supplementary Table S2). The higher *MYB* mRNA expression the lower levels of *CD163*/*CD68* mRNA associations were found also in subgroups with different neoadjuvant chemotherapy (NAC) (Fig. S2). Of note, these results were recapitulated with another dataset (GSE25066)^31^ as shown in Fig. 2b,d, and Supplementary Table S3. Importantly, the negative correlations between *MYB* and *CD68*/*CD163* prevailed across and within subtypes also in patients that did not receive any NAC (Fig. S3). Together these data show that tumors overexpressing *MYB* contain less *CD68*/*CD163* transcript levels independently on the BC subtype.

### TAM recruitment factors are downregulated upon c-Myb overexpression

**Figure 3:**
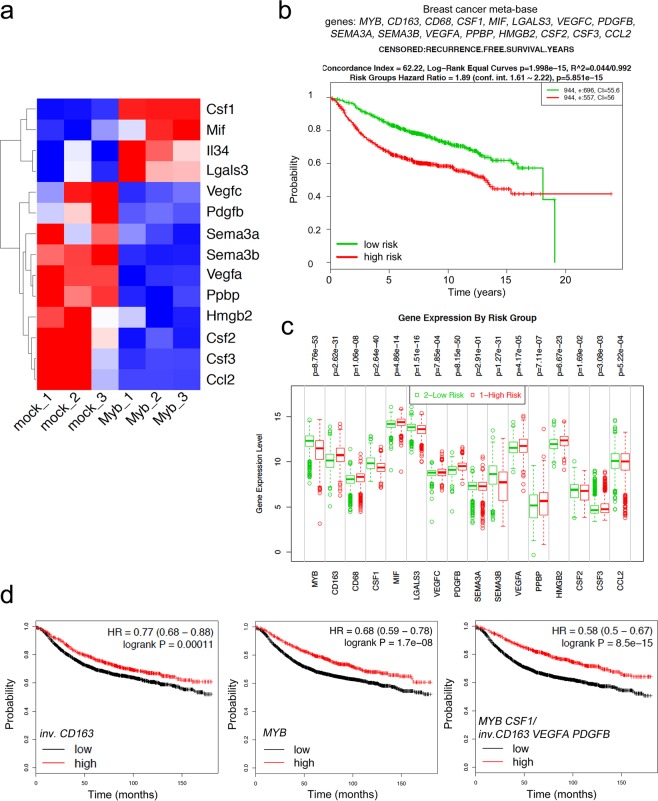
Expression of *MYB* and TAM-related genes is associated with risk of relapse in BCs. (**a**) Heat map of 14 monocyte and macrophage recruitment/migration factors differentially expressed in 4T1 MYB^high^ (Myb1-3) and mock cells as determined by RNA sequencing. (**b**) Kaplan-Meier analysis for MYB-TAM genes (*MYB*, *CD163*, *CD68*, *CSF1*, *MIF*, *LGALS3*, *VEGFC*, *PDGFB*, *SEMA3A*, *SEMA3B*, *VEGFA*, *PPBP*, *HMGB2*, *CSF2*, *CSF3*, *CCL2*) expression under the condition of recurrence free survival in BC patients using SurvExpress database, patients included in Breast Cancer Meta-base split to high (red) and low (green) risk cohorts. (**c**) Expression levels of individual MYB-TAM genes in high vs. low risk groups in Breast Cancer Meta-base. The most significant differentially expressed genes (DEG) are *MYB*, *PDGFB*, *CSF1*, *SEMA3B*, and *CD163*. (**d**) Meta-analyses of BCs patients available on KMplot.com representing the probability of relapse free survival in BCs stratified according to the expression status of *CD163* inverted alone (left), *MYB* alone (middle), and *MYB* in combination with *CSF1* and inverted *CD163*, *VEGFA*, *PDFGB* (right).

There are several cytokines and growth factors known to recruit monocytes/macrophages into tumors^1,32–35^. One of them, chemokine Ccl2 is suppressed by c-Myb in BC cells, as we described previously^23^. However, TAMs utilize multiple chemokine signals to accumulate in the tumor microenvironment, as a single chemokine inhibition does not achieve TAMs depletion^36^. Thus, we took advantage of the established mouse model and screened the c-Myb-responsive transcripts related to TAMs generation/recruitment in 4T1 mammary cancer cells. Comparing transcriptomes of cells overexpressing c-Myb and mock-transfected controls we retrieved the differentially expressed transcripts involved in monocyte/macrophage migration and chemotaxis by gene ontology (GO) terms attribute. A set of 14 potential TAM chemoattractants was identified (Fig. 3a). Besides *Ccl2*, MYB^high^ cells produce less *Csf2*, *Csf3*, *Sema3a*, *Sema3b*, *Vegfa*, *Vegfc*, *Pdgfb*, *Ppbp*, *Hmgb2*, but on contrary more CSF-1R ligands *Csf1* and *IL34*, as well as *Lgals3* and inhibitory factor *Mif* mRNAs when compared to mock-transfected cells (Fig. 3a). Based on these results from a mouse model, we searched human BCs databases. We calculated correlation coefficients for *MYB* and monocyte/macrophage recruitment factors in three independent datasets (Gene Expression Omnibus, GEO, accession numbers GSE22358, GSE12276, GSE25066) and in Medisapiens meta-base. Significant inverse correlations between *VEGFA*, *SEMA3A*, *CSF1*, *CSF2*, *PDGFB* and *MYB* were frequently found, while *SEMA3B* and *MIF* were positively correlated with *MYB* (Supplementary Tables S1–S4). This indicates that c-Myb may suppress TAMs recruitment via regulation of a specific transcription program in BC tumors.

### Expression of MYB/TAM-related genes predicts outcome for BC patients

To investigate the potential prognostic significance of our findings, we researched publicly available platforms of survival analysis, including SurvExpress^37^ and Kaplan-Meier Plotter (KM-Plotter)^38^. To preselect relevant genes within MYB/TAM signature we estimated the prognostic significance of *CD163* and *CD68* in combination with *MYB* and c-Myb-related TAM-recruitment factors (*CCL2*, *CSF2*, *CSF3*, *CSF1*, *VEGFA*, *VEGFC*, *SEMA3A*, *SEMA3B*, *PDGFB*, *PPBP*, *HMGB2*, *LGALS3*, *MIF*, *IL34*) in predicting the recurrence-free survival (RFS) rates using SurvExpress database. SurvExpress implements Cox regression model to estimate β-coefficients of each gene that can be interpreted as a risk coefficients. The prognostic index (PI = β1 × 1 + *β2* × *2* + … + βpxp, where xi is the expression value and the βI is obtained from the Cox fitting), also known as the risk score, is then used to generate risk groups. Overall, the MYB/TAM multigene predictor can significantly separate low- and high-risk groups in the Breast Cancer Meta-base (n = 1888) (Fig. 3b) and in all remaining datasets (n = 19) that monitor recurrence/relapse/metastasis events (Supplementary Table S5). *CD163*, *VEGFA*, *PDGFB*, *CSF1* together with *MYB* were repeatedly found among the most significant differentially expressed genes (DEG) within risk groups (Fig. 3c, Supplementary Table S5). While high-risk groups had higher *CD163*, *VEGFA*, and *PDGFB* expression, they exhibited lower *MYB* and *CSF1* expression levels. In addition, these genes were often among those with significant β-coefficients within the Cox fitting (Supplementary Table S5, significant genes Cox).

Hence, we selected *MYB*, *CSF1*, *CD163*, *VEGFA* and *PDGFB* to compare the probability of RFS in BCs using KM-plotter^38^. BC patients (n = 3951) were stratified according to the expression status of *MYB*, *CSF1* and inverted expression status of *CD163*, *VEGFA* and *PDGFB*. As previously reported by us and others^20,23^ high *MYB* expression decreases the risk of relapse (hazard ratio, HR = 0.68, p = 1.7e − 08) (Fig. 3d). On the other hand, patients with low *CD163* expression had better survival outcome (HR = 0.77, p = 0.00011) that is in line with published data^39^. Interestingly, tumors in the top quartile of *MYB* and *CSF1* expression and the lowest quartile of *CD163*, *VEGFA* and *PDGFB* expression showed better RFS (HR = 0.58, p = 8.5e−15) compared to *MYB* or *CD163* alone (Fig. 3d).

## Discussion

We found previously that c-Myb expression is associated with good prognosis in BC and colorectal cancer patients^23,40,41^. It suppressed a specific subset of inflammatory factors that is elevated in highly metastatic mouse tumors. Blunted inflammatory arsenal in MYB^high^ tumors resulted in severely impaired monocyte-assisted extravasation and lung metastasis^23,42^. Tumor cell-derived inflammatory mediators also shape microenvironment at the tumor site and actively guide infiltration of immune cells from blood^43^. Tumor infiltrating macrophages are often correlated with a bad prognosis, and their abundance relates to the process of metastasis^44,45^. TAMs are key orchestrators of cancer-related inflammation and exert several pro-tumorigenic functions, as they produce a large array of growth factors supporting angiogenesis, and participate in the suppression of the adaptive anti-tumor immune response^46^. Here we explored whether the number of TAMs differs between MYB^high^ and MYB^low^ breast carcinomas. We found that tumors with higher frequency of c-Myb+ tumor cells have indeed lower density of TAMs. These findings suggest that c-Myb-driven transcription changes in tumor cells results in reduced infiltration of the primary tumor by immune cells, which may impair their capacity to form distant metastasis.

In line with previous findings where the presence of TAMs inversely correlated with ER expression in BC^10,47^, we observed more CD68+ cells in triple-negative breast tumors compared to luminal subtypes. Medrek *et al*. also described the tumor stroma of luminal subtypes to be rarely infiltrated by TAMs that is in contrary to the densely infiltrated tumors of the basal-like subtype^29^. The subtype-dependent TAMs abundance was confirmed with microarray expression data showing that the basal-like breast cancer had significantly higher levels of *CD163* and *CD68* mRNAs compared to luminal breast cancer^29^. Of note, high *MYB* levels are generally found in luminal subtypes^23,48^. In this study, we evaluated mRNA levels of the two macrophage markers, CD68 as a pan-macrophage marker and CD163 specific for M2-like macrophages^49,50^, in large cohorts of BC patients to confirm our immunohistochemical findings. Human microarray data mining showed that expression of both *CD68* and *CD163* often inversely correlates with *MYB*, even within molecular subtypes, implying that c-Myb reduces TAMs in BCs independently on the ER-status.

An array of tumor-derived chemoattractants such as CSF1, CSF2, CCL2, VEGFA, and SEMA3A contribute to the recruitment of monocytic precursors, resulting in TAMs accumulation^1,32–34,51–53^. A prominent monocyte recruiting factor Ccl2 is one of the inflammatory mediators directly repressed by c-Myb^23,54^. The correlation between macrophage accumulation and Ccl2 expression has been demonstrated in breast carcinomas and Ccl2 neutralization was found to attenuate recruitment of inflammatory monocytes and reduce metastasis formation in tumor-bearing mice^26^. A set of multiple cytokines shape abundance and phenotype of macrophages in a tumor, rather than a single ligand-receptor pair^36^. We hypothesized that decreased accumulation of TAMs in MYB^high^ tumors is caused by downregulation of several factors, including *SEMA3A*, *VEGFA*, *CSF2* or *PDGFB* that we found to be inversely correlated with c-Myb not only in mammary cell lines but also in primary human BCs. However, reduced amount of TAMs associated with high *MYB* expression by tumor cells may also arise from altered TAMs proliferation not only recruitment of precursors from circulation. Recent evidence indicates that fully differentiated macrophages may proliferate *in situ*, thus increasing the pool of TAMs^55^. Interestingly, the cell-surface guidance molecule SEMA3A contributes to differential proliferative control of TAMs^56^. Dissecting the mechanism of c-Myb effect on TAMs density in BCs that likely involves a specific transcription module regulation, requires further functional studies. How are tumors with high *MYB* expression populated by TAMs, whether their recruitment, or proliferation is altered by tumor-derived cytokine network under control of c-Myb, whether such milieu could be mimicked by gene depletion/overexpression or pharmacologically, and how TAMs differ in their properties (not only quantity) in Myb-high vs. Myb-low tumors etc. should be investigated in preclinical models.

The presence of proliferating TAMs in human BC is associated with poor clinical outcomes and early recurrence^10^. Moreover, the expression of the macrophage antigen CD163 in BCs has a prognostic impact on the occurrence of distant metastases and reduced patient survival time^29,39^ that we also confirmed using publically available expression databases. Combination of *MYB*, its potential target genes and *CD163* enhanced the relapse risk assessment compared to *CD163* alone, and *vice versa*, a biomarker consisting of *MYB* and TAM-related transcripts *CD163*, *CSF1*, *VEGFA* and *PDGFB* increased the prognostic performance of *MYB*. These results indicate a clinical relevance of the identified MYB-TAMs liaison.

Unexpectedly, we found that *CSF1* expression, unlike *CD163*, *VEGFA* and *PDGFB*, is significantly higher in low-risk group. CSF-1 was one of the TAM-related cytokines up-regulated in MYB^high^ 4T1 cells, along with interleukin 34 (IL34), but correlation analyses in clinical samples mostly revealed negative association. CSF-1 is a myelopoietic growth factor regulating the recruitment, proliferation, differentiation, and survival of macrophages via binding to CSF-1 receptor (CSF-1R/c-Fms/CD115). Interestingly, IL34 is a newly discovered alternative ligand for CSF-1R, triggering the same signaling events and promoting the differentiation and survival of macrophages, only with different polarization potential^57^. The role of CSF1/CSF-1R as predictive factors in BC remains unclear. Although frequently referred as a poor prognosis factors, clinical evidence shows variable associations that rather depend on patient groups or protein localization^58–64^. Underscoring the complexity of CSF-1/CSF-1R pair in BC prognosis, Beck *et al*. found that a CSF-1 response signature predicted different outcomes for patients with breast cancer depending on the tumor subtype^65^.

The function of CSF1/CSF-1R remains controversial also in experimental models of breast cancer. In macrophage-deficient MMTV-PyMT (mouse mammary tumor virus- polyoma middle T-antigen) mice carrying a null mutation in the *Csf1* gene, TAMs were unable to accumulate in primary tumors and resulted in reduced lung metastasis^66^. Macrophage-depletion mimics *Csf1* deficiency in reduced lung metastatic seeding^67^. In MCF-7 mammary carcinoma cell xenografts CSF-1 block has been shown to reduce host macrophage infiltration and suppress tumor growth^68^. This, along with other observations of the beneficial effects of targeting CSF-1R in various cancers^69–71^, has led to the initiation of several clinical trials with either a monoclonal antibody or a small molecule inhibitor of CSF-1R^72^. However, recent studies placed a cautionary note on blocking CSF-1 signaling as a therapeutic modality in cancer. Neutralizing anti–CSF-1R and anti–CSF-1 antibodies, or small-molecule inhibitors of CSF-1R, not only left the tumor growth unaffected but actually increased spontaneous metastasis^63^. The block of CSF-1R or CSF-1 led to increased levels of serum G-CSF (granulocyte colony stimulating factor, CSF-3), increased frequency of neutrophils, while TAMs were variably reduced. Block of G-CSF receptor overcomes the increase in metastasis and neutrophil numbers, indicating that this enhanced metastasis is driven by G-CSF that in turn alters the phenotype of TAMs^35,63^. Of note, *CSF1* is one of the genes included in lung-metastatic signature that is down-regulated in aggressive metastatic MDA-MB-231 cells^73^. Whether differential control CSF-1/CSF-3 by c-Myb may guide specific leukocyte infiltrate that accounts for lung metastasis suppression requires further investigations.

Because high TAM infiltration is associated with poor prognosis and therapeutic failure in cancer patients, inhibition of recruitment and retention of macrophages may represent a valuable strategy to combine with conventional therapies. It is plausible that TAMs utilize multiple signals to accumulate in the tumor microenvironment, which makes any approach to eliminate them difficult. Identification of a transcription program running in tumor cells with high *MYB* expression that prevents macrophage accumulation may open new venues towards better prognosis estimation and potentially towards TAMs targeting.

## Methods

### Immunohistochemistry

The study group included 86 breast cancer patients (median age 53 years) with invasive breast cancer who had undergone surgical treatment at Lviv Regional Oncological Center in the period 2013–2016 (Table S6). All tumor samples were obtained as surgical specimen before any kind of treatment. This study complied with the standards of the Declaration of Helsinki and guidelines for tumor marker prognostic studies (REMARK)^74^ and was approved by the Ethical Committee of Lviv Regional Oncological center. Informed consent was obtained from all individual participants included in the study.

IHC detection of c-Myb, CD68, ER, PgR, HER2 was performed on formalin fixed paraffin embedded tissues of primary tumors as described previously^23^. Briefly, 4 μm thick tissue sections were deparaffinized, rehydrated and incubated in 3% Hydrogen Peroxide for 5 minutes. The antigen retrieval was performed by heating the sections in citrate buffer (pH 6.0). The slides were blocked for 5 minutes with Ultra V Block solution from UltraVision LP Large Volume Detection System HRP (Horseradish Peroxidase) Polymer Ready-To-Use kit (Thermo Fisher Scientific, UK), and incubated with the primary antibodies: rabbit monoclonal anti-c-Myb (clone EP769Y, dilution 1:100, Abcam, Cambridge, UK) at room temperature (RT) overnight; rabbit monoclonal anti-CD68 (clone KP1, dilution 1:200, Thermo Fisher Scientific, UK), rabbit monoclonal anti-ER (clone SP1, dilution 1:200; Thermo Fisher Scientific, UK), rabbit monoclonal anti-PgR (clone SP2, dilution 1:200; Thermo Fisher Scientific, UK) at RT 30 min, and rabbit monoclonal anti-HER2 (clone SP3, dilution 1:350; Thermo Fisher Scientific, UK) at RT 20 min.

The slides were washed 4 times in phosphate-buffered saline (PBS) and incubated in Primary Antibody Enhancer (from UltraVision kit) at RT for 10 min. Then, the slides were incubated with HRP Polymer (from UltraVision kit) at RT for 15 minutes, washed 4 times in PBS and incubated with 3,3΄-diaminobenzidine (DAKO, Glostrup, Denmark) as chromogen for 5 min. The slides were counterstained with Mayer’s hematoxylin solution (Sigma Aldrich). Negative controls were prepared by incubating samples in the absence of a primary antibody. Evaluation of all IHC results was performed using a uniform Zeiss microscope independently by two pathologists.

The tumors were evaluated for percentage of immunostained positive cells in 10 random fields at magnification x200. The amount of tumor infiltrating macrophages was evaluated as positive cells at x1350 magnification (in 20 stromal and 20 tumor fields), which gave the total amount of macrophages in 40 high power fields. This amount has been used for all further calculations.

### Expression of TAM recruitment factors in MYB^high^ mammary cancer cell line

Derivation and RNA sequencing (RNAseq) of 4T1 cells overexpressing *Myb* (MYB^high^) were described previously^23^. The expression levels of potential TAM recruitment factors were analyzed as follows: differentially expressed genes in MYB^high^ and mock-transfected cells were searched for GO terms associated with monocyte and macrophage migration/activation/chemotaxis (GO:0042116, GO:0048246, GO:0002548, GO:0042056, GO:1905517, GO:0002688). Out of 22 genes associated with these GO terms, 14 were selected encoding potential paracrine factors directed towards macrophages. These differentially expressed transcripts were clustered and shown in heatmap using FGCZ (Functional Genomics Center Zürich) Heatmap tool (http://fgcz-shiny.uzh.ch). The RNAseq data are available in Gene Expression Omnibus (GEO, NCBI) under the accession number GSE104264.

### Correlation analysis

We used Medisapiens (ist.medisapiens.com) and NCBI GEO databases to assess the differential expression of *MYB*, *CD68*, *CD163*, *CSF1*, *CSF2*, *CSF3*, *PDGFB*, *SEMA3A*, *SEMA3B*, *VEGFA*, *VEGFC*, *PPBP*, *MIF*, *HMBG2*, *IL34*, *LGALS3* mRNA in human BCs. Correlations between *MYB* and *CCL2* were shown previously^23^. Pearson correlations were calculated with the GraphPad Prism software (version 6.07). Besides Medisapiens, three independent GEO datasets were used (accession numbers: GSE25066, GSE22358, GSE12276), results in Fig. 2 and Supplementary Tables S1–S4.

### Survival analysis

To assess the prognostic significance of a list of MYB-TAMs related genes we used SurvExpress database^37^. All datasets offering recurrence, relapse or metastasis endpoints were used for Cox fitting, the maximum row average for duplicated genes, two risk groups split at the median prognostic index. The log-rank test was used to evaluate statistically the equality of survival curves. All results are summarized in Supplementary Table S5.

Kaplan–Meier plots representing the probability of RFS in BCs stratified according to the expression status of *MYB*, *CSF1*, *CD163*, *PDGFB* and *VEGFA* were calculated with KM-plotter (kmplot.com)^38^. Follow-up threshold set for 15 years, patients were split by upper quartile expression, only JetSet best probe set per gene included, the expression of *CD163*, *PDGFB*, and *VEGFA* was inverted. The log-rank test was used to assess the significance of the correlation between gene(s) expression and shorter survival outcome.

### Statistics

Statistical analysis was performed with the GraphPad Prism software (version 6.07). For correlation analysis Pearson correlation coefficients were calculated. Survival curves were evaluated using the log-rank test.

## Supplementary information

Supplementary information

## Data Availability

The RNAseq data are available in Gene Expression Omnibus (GEO, NCBI) under the accession number GSE104264. Other datasets generated and analysed during the current study are available from the corresponding author on reasonable request.
