# Supplementary material for: Low infiltration of tumor-associated macrophages in high c-Myb-expressing breast tumors
Source: Sci Rep. 2019 Aug 12;9:11634. doi: 10.1038/s41598-019-48051-1 (PMC6690941; doi:10.1038/s41598-019-48051-1)
Supplement: Supplementary file 1 — Supplementary information [file 41598_2019_48051_MOESM1_ESM.pdf]

Supplementary information

Volodko N, Gutor T, Petronchak O, Huley R, Dúcka M, Šmarda J, Borsig L, Beneš P, Knopfová L

Low infiltration of tumor-associated macrophages in high c-Myb-expressing breast tumors

Figure S1. Correlation between frequency of c-Myb+ tumor cells and number of CD68+ cells as determined by IHC in BC patients stratified according to the ER expression. Correlations in groups of (A) 66 ER+ patients, (B) 20 ER- patients. Pearson correlation coefficient (r), logrank p value (p) and number of patients (n) are indicated.

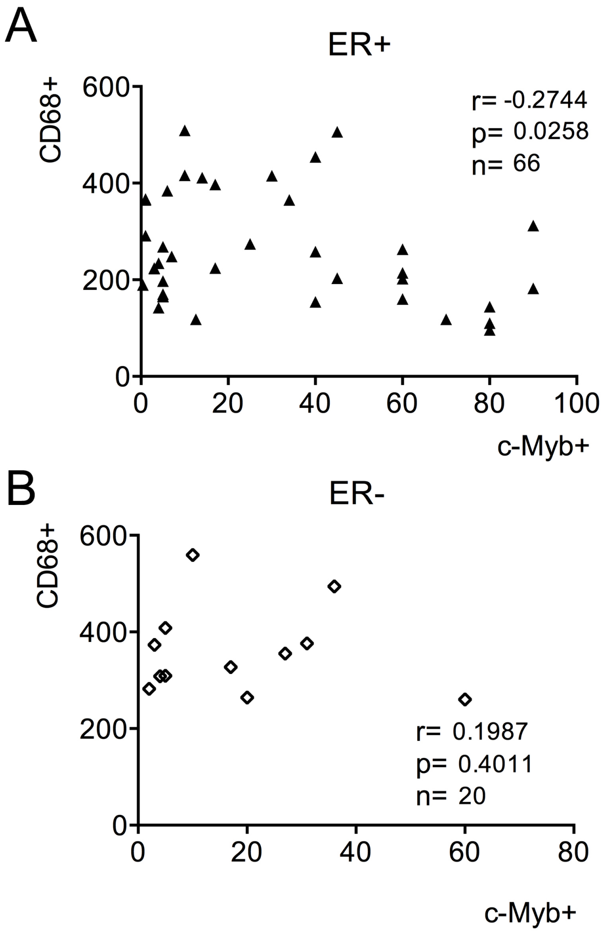

Figure S2. *MYB* mRNA levels are inversely correlated with *CD68* and *CD163* mRNAs in human BCs in subgroups with different neoadjuvant chemotherapy (NAC). GSE22358 dataset was used for correlation analysis as in Fig 2. Patients were stratified according to the received NAC in 2 groups: docetaxel-Capecitabine with **(a)** and **(b)** without Trastuzumab. Pearson correlation coefficients (r) between mRNA expression of indicated genes (*MYB* vs *CD68*, *MYB* vs *CD163*), logrank p value and number of patients (n) are indicated in the graphs.

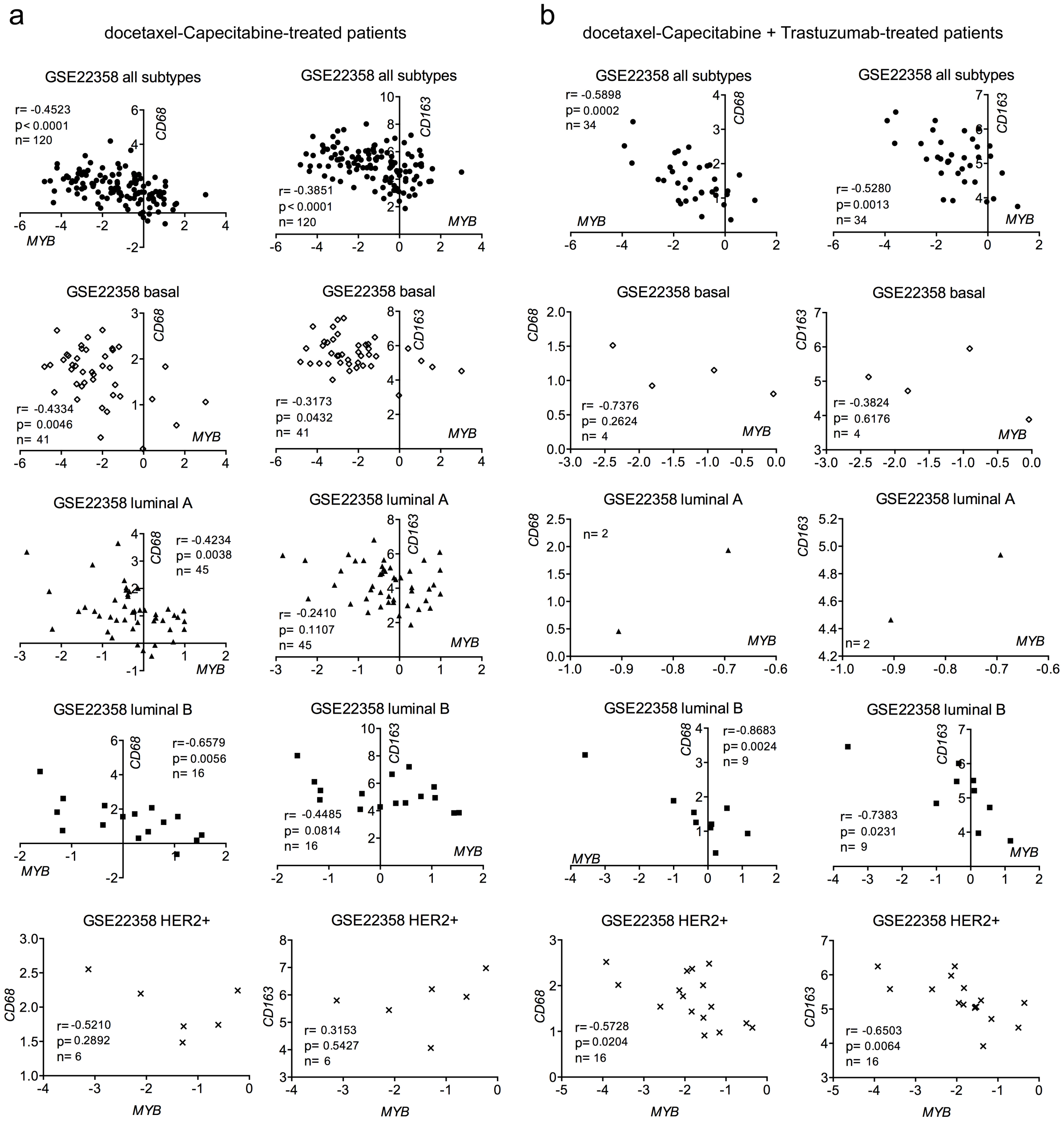

Figure S3. Inverse correlations between *MYB* and *CD68/CD163* mRNA levels do not result from chemotherapy. GSE21974 dataset was used for correlation analysis (Stickeler *et al*., Oncol Rep. 2011). The cohort consists of 32 women with primary invasive breast cancer with dual tumor specimens obtained before and after 4 cycles of NAC. Samples obtained before and after NAC were analyzed separately **(a)**. Subtype categories basal and non-basal were applied according to the original study **(b)**. Pearson correlation coefficients (r) between mRNA expression of indicated genes (*MYB* vs *CD68*, *MYB* vs *CD163*), logrank p value and number of patients (n) are indicated in the graphs.

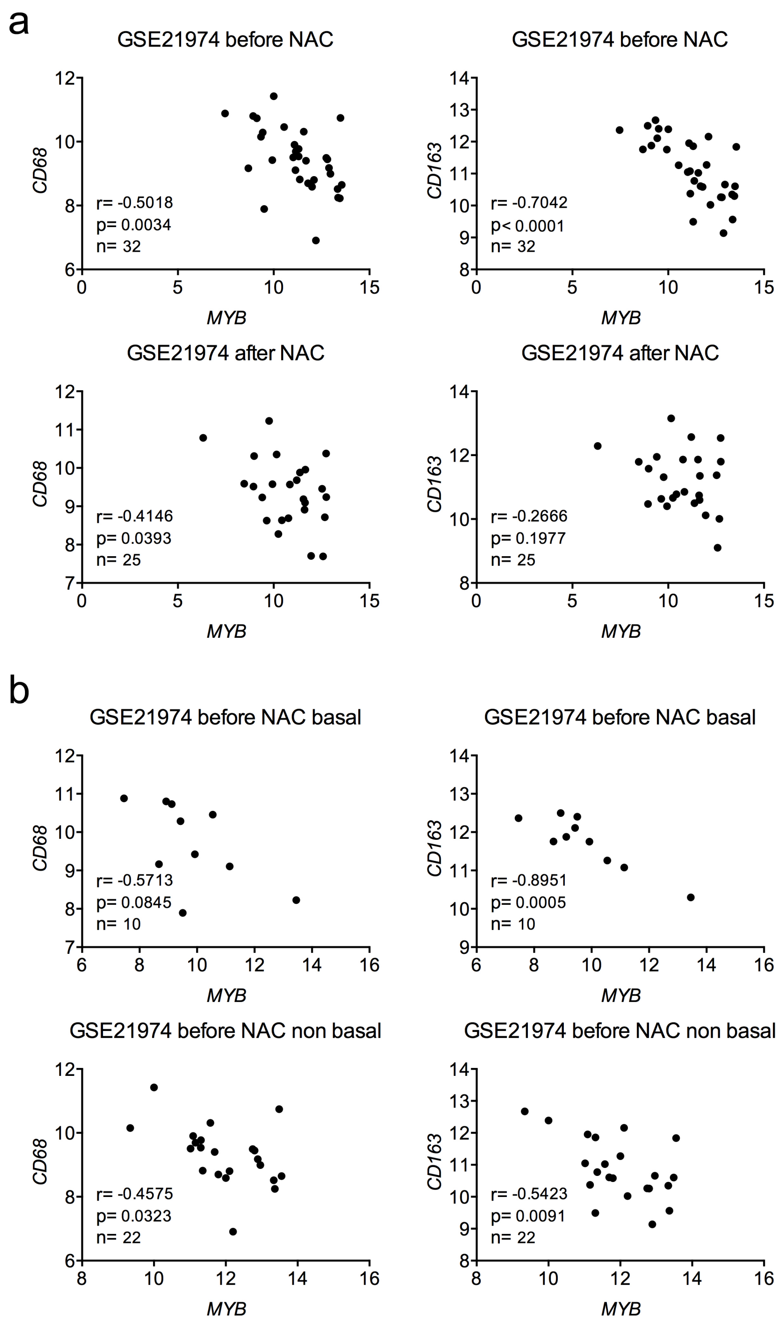

Table S1. Correlations of mRNA expression levels of *MYB* and TAM-associated proteins as retrieved from Medisapiens database. Medisapiens (http://ist.medisapiens.com). query filter: breast carcinomas, other; breast lobular carcinomas, breast ductal carcinomas. Pearson correlation coefficient (r), logrank p value (p) and number of patients (n) are indicated.

	breast carcinoma. other			breast lobular carcinoma			breast ductal carcinoma		
MYB/		p	n		p	n		p	n
CD68	**-0.09**	<0.001	1830	**-0.282**	<0.001	83	**-0.077**	<0.001	645
CD163	**-0.192**	<0.001	1830	**-0.194**	<0.001	96	**-0.08**	<0.001	720
CSF1	**-0.199**	<0.001	1830	**-0.266**	<0.001	96	**-0.166**	<0.001	720
CSF2	**-0.137**	<0.001	1830	**-0.082**	<0.001	96	**-0.039**	<0.001	720
CSF3	**-0.025**	<0.001	1830	**-0.182**	<0.001	96	**-0.062**	<0.001	720
HMGB2	**-0.125**	<0.001	1830	**0.023**	0.83	96	**-0.132**	<0.001	720
IL34	**-0.189**	<0.001	540	**-0.083**	<0.001	80	**-0.079**	<0.001	557
LGALS3	**-0.036**	<0.001	1830	**-0.169**	<0.001	96	**-0.066**	<0.001	720
PDGFB	**-0.043**	<0.001	1830	**-0.29**	<0.001	96	**-0.097**	<0.001	720
PPBP	**-0.116**	<0.001	1830	**-0.336**	<0.001	96	**-0.084**	<0.001	720
SEMA3A	**-0.118**	<0.001	1830	**-0.173**	<0.001	96	**-0.09**	<0.001	720
SEMA3B	**0.228**	<0.001	1830	**0.068**	0.51	96	**0.221**	<0.001	720
VEGFA	**-0.257**	<0.001	1830	**-0.016**	0.88	96	**-0.128**	<0.001	720
VEGFC	**-0.219**	<0.001	1830	**-0.096**	<0.001	96	**-0.079**	<0.001	720

Table S2. Correlations of mRNA expression levels of *MYB* and TAM-associated proteins in GEO dataset GSE22358.
Pearson correlation coefficient (r), logrank p value (p) and number of patients (n) are indicated in all patients and subgroups of basal, luminal A, luminal B and HER2+ tumors. Probe ID indicated for each gene.

**Glück Breast GSE22358**	**all subtypes (n=152)**			**basal (n=45)**			**luminal A (n=46)**			**luminal B (n=25)**			**HER2+ (n=21)**		
**MYB / 9297**	r	p (two-tailed)		r	p (two-tailed)		r	p (two-tailed)		r	p (two-tailed)		r	p (two-tailed)	
CD163 / 42516	-0.3998	< 0.0001	****	-0.3418	0.0216	*	-0.2459	0.0957	ns	-0.5012	0.0107	*	-0.2181	0.3294	ns
CD68 / 38360	-0.4702	< 0.0001	****	-0.4644	0.0013	**	-0.4099	0.0042	**	-0.6897	0.0001	***	-0.4809	0.0235	*
CSF1 / 15024	-0.3126	< 0.0001	****	-0.3206	0.0318	*	-0.0707	0.6405	ns	-0.1895	0.3643	ns	-0.2256	0.3255	ns
CSF2 / 15856	-0.2237	0.0056	**	-0.1687	0.2681	ns	-0.0082	0.9564	ns	-0.3307	0.1064	ns	0.1104	0.6339	ns
CSF3 / 23682	-0.009	0.91	ns	-0.1156	0.4495	ns	-0.2151	0.1464	ns	-0.3468	0.0895	ns	-0.2607	0.2413	ns
HMGB2 / 18601	0.0932	0.2504	ns	0.4547	0.0017	**	0.3637	0.012	*	-0.05293	0.8016	ns	0.4275	0.0472	*
LGALS3 / 14190	-0.2278	0.0045	**	-0.4235	0.0037	**	-0.3497	0.016	*	-0.3418	0.0944	ns	0.2462	0.2695	ns
MIF / 2289	0.1325	0.1014	ns	0.0425	0.7818	ns	0.0525	0.7262	ns	0.3904	0.0537	ns	0.1068	0.6362	ns
PDGFB / 8181	-0.2217	0.006	**	-0.2556	0.0901	ns	-0.3648	0.0127	*	-0.1103	0.5997	ns	-0.1913	0.4063	ns
PPBP / 10023	-0.1241	0.1252	ns	-0.2493	0.0986	ns	-0.0908	0.5439	ns	-0.1664	0.4267	ns	0.2257	0.3126	ns
SEMA3A / 17255	-0.3956	< 0.0001	****	-0.2565	0.089	ns	-0.4699	0.001	***	-0.6502	0.0004	***	-0.2146	0.3502	ns
SEMA3B / 26742	0.3329	< 0.0001	****	-0.2994	0.0457	*	0.3451	0.0188	*	0.5253	0.007	**	-0.0025	0.9914	ns
VEGFA / 5470	-0.4317	< 0.0001	****	-0.3807	0.0099	**	-0.2665	0.0734	ns	0.0442	0.8338	ns	-0.1703	0.4606	ns
VEGFC / 4550	-0.406	< 0.0001	****	-0.5372	0.0001	**	-0.4292	0.0026	**	-0.4668	0.0187	*	-0.4329	0.0442	*

Table S3. Correlations of mRNA expression levels of *MYB* and TAM-associated proteins in GEO dataset GSE25066.
Pearson correlation coefficient (r), logrank p value (p) and number of patients (n) are indicated in all patients and subgroups of basal, luminal A, luminal B and HER2+ tumors. Probe ID indicated for each gene.

**Hatzis Breast (GSE25066)**	**all subtypes (n=508)**			**basal (n=189)**			**luminal A (n=160)**			**luminal B (n=78)**			**HER2+ (n=37)**		
MYB / 204798_at	r	p (two-tailed)		r	p (two-tailed)		r	p (two-tailed)		r	p (two-tailed)		r	p (two-tailed)	
CD163 / 215049_x_at	-0.4446	< 0.0001	****	-0.298	< 0.0001	****	-0.3614	< 0.0001	****	-0.1864	0.1023	ns	-0.3396	0.0397	*
CD68 / 203507_at	-0.2216	< 0.0001	****	-0.1303	0.0739	ns	-0.1821	0.0212	*	-0.1378	0.229	ns	-0.3178	0.0553	ns
CSF1 / 209716_at	-0.1447	0.0011	**	-0.156	0.0323	*	-0.1776	0.0252	*	0.04631	0.6873	ns	0.09919	0.5592	ns
CSF2 / 210229_s_at	-0.2305	< 0.0001	****	-0.025	0.7338	ns	-0.4268	< 0.0001	****	-0.3005	0.0075	**	-0.1384	0.4139	ns
CSF3 / 207442_at	-0.0165	0.7103	ns	0.0569	0.4371	ns	-0.1124	0.1569	ns	0.03388	0.7684	ns	-0.1317	0.437	ns
HMGB2 / 208808_s_at	-0.1379	0.0018	**	0.1651	0.0232	*	0.03504	0.66	ns	0.003653	0.9747	ns	0.2828	0.0899	ns
LGALS3 / 208949_s_at	-0.1172	0.0082	**	-0.0689	0.3464	ns	0.05573	0.484	ns	-0.06668	0.5619	ns	-0.319	0.0543	ns
MIF / 217871_s_at	0.1887	< 0.0001	****	0.2927	< 0.0001	****	0.18999	0.0161	*	0.3396	0.0024	**	0.2051	0.2233	ns
PDGFB / 216055_at	-0.1224	0.0058	**	-0.078	0.2885	ns	-0.3291	< 0.0001	****	-0.1776	0.1197	ns	-0.02678	0.875	ns
PPBP / 214146_s_at	-0.0651	0.1426	ns	-0.1052	0.15	ns	-0.1554	0.0498	*	-0.07865	0.4937		-0.1086	0.5221	ns
SEMA3A / 206805_at	-0.2657	< 0.0001	****	-0.135	0.0636	ns	-0.4756	< 0.0001	****	-0.264	0.0195	*	-0.1914	0.2564	ns
SEMA3B / 203071_at	0.4415	< 0.0001	****	-0.072	0.3275	ns	0.2805	0.0003	***	0.3677	0.0009	***	0.4516	0.005	**
VEGFA / 210513_s_at	-0.201	< 0.0001	****	-0.075	0.3054	ns	0.06322	0.4285	ns	0.1084	0.3447	ns	0.2083	0.2161	ns
VEGFC / 209946_at	-0.0796	0.0732	ns	-0.045	0.539	ns	-0.0666	0.403	ns	-0.4318	< 0.0001	****	-0.306	0.0655	ns

Table S4. Correlations of mRNA expression levels of *MYB* and TAM-associated proteins in GEO dataset GSE12276.
Pearson correlation coefficient (r), logrank p value (p) and number of patients (n) are indicated in all patients. Probe ID indicated for each gene.

**Boss Breast (GSE12276)** MYB / 204798_at	**all subtypes (n=204)**		
	r	p (two-tailed)	
CD163 / 215049_x_at	-0.30623	< 0.0001	****
CD68 / 203507_at	-0.02868	0.6839	ns
CSF1 / 209716_at	-0.2158	0.002	**
CSF2 / 210229_s_at	-0.07556	0.2828	ns
CSF3 / 207442_at	0.05056	0.4888	ns
HMGB2 / 208808_s_at	-0.16334	0.0196	*
LGALS3 / 208949_s_at	-0.04118	0.5587	ns
MIF / 217871_s_at	0.05613	0.4252	ns
PDGFB / 216055_at	-0.05729	0.4157	ns
PPBP / 214146_s_at	-0.0962	0.1711	ns
SEMA3A / 206805_at	-0.2422	0.0005	***
SEMA3B / 203071_at	0.3759	< 0.0001	****
VEGFA / 210513_s_at	-0.2327	0.0008	***
VEGFC / 209946_at	-0.1193	0.0892	ns

Table S5. Prognostic significance of *MYB, CD163, CD68, CCL2, CSF2, CSF3, CSF1, VEGFA, VEGFC, SEMA3A, SEMA3B, PDGFB, PPBP, HMGB2, LGALS3, MIF, IL34* in SurvExpress datasets. CI = Concordance Index, DEG = Differentially Expressed Genes

	n	Censored	CI	logrank p	Significant genes (Cox)	Top5 significant DEG
Breast cancer recurrence	1561	Recurrence free survival	0.63	6.335e-13	*CSF1, PDGFB, CD163, CCL2, MIF*	*CSF1, PDGFB, HMGB2, MIF, VEGFA*
Breast cancer meta-base	1888	Recurrence free survival	0.62	1.998e−15	*CSF1, PDGFB, CD163, CCL2, MYB*	*MYB, PDGFB, CSF1, SEMA3B, CD163*
van´t Veer - Van De Vijver Nature 2002	295	Recurrence years	0.70	6.185e−11	*MYB, VEGFA*	*MYB, VEGFA, PDGFB, HMGB2, CD163*
Sotiriou Van de Vijver Breast GSE2990	187	Relapse free survival	0.71	0.0001795	*CSF1, PDGFB, CD163*	*CSF1, HMGB2, LGALS3, VEGFA, CD163*
Loi Sotiriou Breast GSE6532	225	Relapse free survival	0.71	1.868e-09	*CCL2, PDGFB*	*PDGFB, CSF2, MIF, CCL2, SEMA3A*
Kao Huang Breast GSE20685	327	Metastasis months	0.69	7.857e-05	*CSF1, VEGFC*	*VEGFC, MYB, CSF1, CSF3, VEGFA*
Ivshina Miller Breast GSE4922	249	Relapse free survival	0.67	6.783e-05	*CD163, CSF1, HMGB2*	*CSF1, HMGB2, MIF, VEGFC, VEGFA*
Pawitan Breast GSE1456	159	Relapse free survival	0.72	0.0004465	*CD68, CSF1, PDGFB*	*HMGB2, MIF, CD163, PDGFB, CSF1*
Zhang Foekens Breast GSE12093	136	Relapse free survival	0.78	0.0007319	*MYB, CD163, PDGFB, VEGFC*	*VEGFC, HMGB2, CD163, CSF3, MYB*
Bos Massague Breast GSE12276	204	Relapse brain	0.90	1.989e-05	*MYB, CD163, CSF1, PPBP, PDGFB, VEGFC*	*MYB, CD163, SEMA3B, PPBP, CCL2*
Zhuo-Benz Breast GSE7378	54	Recurrence event	1	0.00114		*CD163, HMGB2, VEGFC, PPBP, CSF1*
Wang Richardson Breast GSE19615	115	Relapse months	0.83	0.0006324	*VEGFA, SEMA3B*	*SEMA3B, VEGFA, CCL2, VEGFC, CSF2*
Chin Gray Breast E-TABM-158	130	Distant relapse months	0.78	0.0001275	*CD163, SEMA3B, SEMA3A, CCL2*	*PDGFB, HMGB2, SEMA3A, CCL2, VEGFA*
Vincent Darbon Breast GSE9893	155	Distant recurrence	0.83	3.144e-05	*CD68, CSF1, PPBP, LGALS3*	*SEMA3A, PPBP, MYB, CSF3, MIF*
Prat-Perou-Breast-GSE18229	254	RFS	0.71	3.203e-07	*MYB, SEMA3B, LGALS3*	*SEMA3B, MYB, LGALS3, PDGFB, CD163*
Wang Foekens Minn Massague Breast GSE5327	58	Metastasis free survival	0.83	0.01883	*PDGFB*	*CD163, PDGFB, CD68, VEGFC, MIF*
Wang Leong Breast GSE45725	340	Recurrence months	0.65	9.378e-12	*MYB, VEGFA, LGALS3, PDGFB*	*LGALS3, MYB, PDGFB, VEGFA, CSF3*
Desmedt Sotiriou Breast GSE7390	189	Relapse free survival	0.62	0.001636	*LGALS3*	*VEGFA, CD68, CD163, MYB, CSF1*
Desmedt Sotiriou Breast GSE16391	55	Relapse free survival	0.72	1.371e-06	*CD68*	*CD68, PDGFB, SEMA3B, SEMA3A, CSF2*
Loi Sotiriou Breast GSE9195	77	Relapse free survival	0.85	0.004909	*MYB, SEMA3B, CCL2*	*MYB, SEMA3B, CSF3, CD68, PPBP*

Table S6. Patients' characteristics

Characteristics	No of patients<br>86 (100%)
**Median age, years** (range)	53 (31-84)
**Primary tumor size, cm**	
≤2	44 (51%)
>2 to≥ 5	39 (46%)
>5	3 (3%)
**Lymph nodes involvement**	
negative	38 (44%)
positive	48 (56%)
**Histologic grade**	
1	13 (15%)
2	51 (59%)
3	22 (26%)
**Tumor molecular subtypes**	
Luminal A	46 (54%)
Luminal B	20 (23%)
Her2+	8 (9 %)
Triple negative	12 (14%)
**Local treatment**	
Breast conserving surgery + radiation therapy	21 (24%)
Breast conserving surgery without radiation therapy	11 (13%)
Mastectomy	39 (45,5%)
Mastectomy + radiation therapy	15 (17,5%)
